# Multifunctional Ionic Polymers from Deep Eutectic Monomers Based on Polyphenols

**DOI:** 10.1021/acsmacrolett.2c00657

**Published:** 2023-01-12

**Authors:** Jon López de Lacalle, Antonela Gallastegui, Jorge L. Olmedo-Martínez, Melissa Moya, Naroa Lopez-Larrea, Matías L. Picchio, David Mecerreyes

**Affiliations:** † POLYMAT University of the Basque Country UPV/EHU, Paseo Manuel de Lardizábal, 3, 20018 Donostia-San Sebastián, Spain; ‡ Laboratorio de Investigación, Universidad de Ciencias Médicas, 10108 San José, Costa Rica; § Facultad de Microbiología, Universidad de Ciencias Médicas, 10108 San José, Costa Rica; ∥ Instituto de Desarrollo Tecnológico para la Industria Química (INTEC), CONICET, Güemes 3450, Santa Fe 3000, Argentina; ⊥ IKERBASQUE, Basque Foundation for Science, Plaza Euskadi 5, 48009 Bilbao, Spain

## Abstract

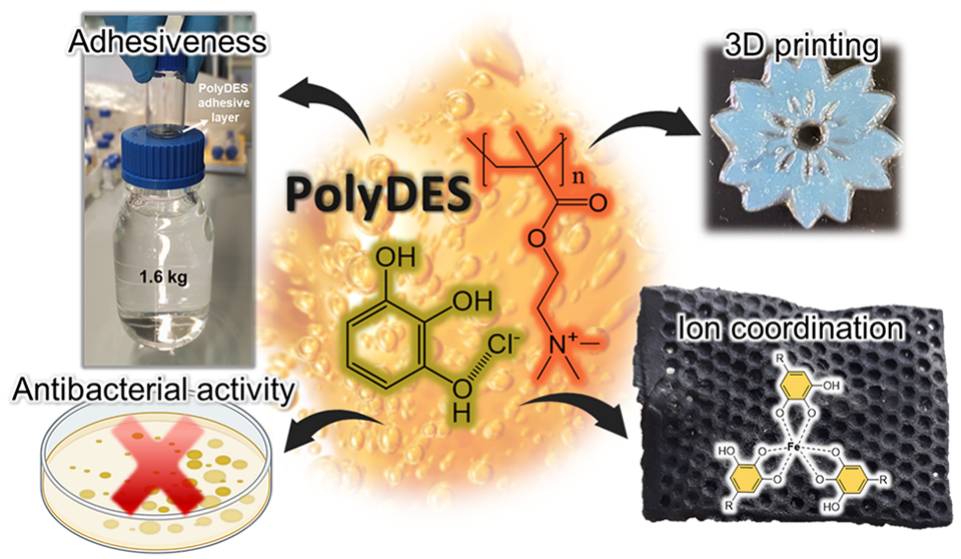

Herein we report a novel family of deep eutectic monomers and the corresponding polymers, made of (meth)acrylic ammonium salts and a series of biobased polyphenols bearing catechol or pyrogallol motifs. Phenolic chemistry allows modulating molecular interactions by tuning the ionic polymer properties from soft adhesive to tough materials. For instance, pyrogallol and hydrocaffeic acid-derived ionic polymers showed outstanding adhesiveness (>1 MPa), while tannic acid/gallic acid polymers with dense hydrogen bond distribution afforded ultratough elastomers (stretchability ≈1000% and strength ≈3 MPa). Additionally, phenolic polymeric deep eutectic solvents (polyDES) featured metal complexation ability, antibacterial properties, and fast processability by digital light 3D printing.

Deep eutectic solvents (DES) have attracted great interest in the past decade as green and low-cost solvents in high-demanding applications.^1^ DES are expected to substitute classic ionic liquids (ILs) due to their easier preparation and possibilities for biosources. DES solvents are mixtures of two or more pure compounds for which the eutectic point temperature is significantly below that of an ideal liquid mixture.^2^ These negative deviations from ideality are often caused by strong interactions between the usually solid DES components, called hydrogen bond donor (HBD) and hydrogen bond acceptor (HBA).^3^

DES chemistry is also being applied to the design of new ionic polymers.^4,5^ In particular, deep eutectic monomers (DEMs) that are polymerizable DES and the resultant polyDES have stepped into the spotlight of materials science. Traditional DEMs rely on mixtures of hydrogen bond donor monomers such as acrylic acid,^6−9^ methacrylic acid,^10,11^ and acrylamide,^12−14^ among others,^15,16^ with HBA ammonium salts. As one example, these polyDES have recently been reported as conductive elastomers and strain sensors.^17−22^ The second class of DEMs consists of polymerizable ammonium salts (e.g., cholinium bromide and chloride-derived (meth)acrylates) and proper HBDs, like organic acids and urea.^23,24^ Regrettably, both types of DEMs show limited functional features beyond their intrinsic ionic conductivity.^25^ Therefore, innovative active components are being fervently sought for multifunctional material design to broaden the application scope of this emerging class of ionic polymers.

This letter presents a fascinating set of DEMs where natural polyphenols have been introduced as multifunctional HBDs in combination with methacrylic and acrylic quaternary ammonium monomers. The evaluated phenolic derivatives such as pyrogallol (PGA), tannic acid (TA), gallic acid (GA), protocatechuic acid (PA), hydrocaffeic acid (HCA), and vanillyl alcohol (VA) showed strong interactions with both ammonium (meth)acrylic monomers resulting in complete suppression of the mixture melting point. The chemical structures and respective acronyms of the DEM and polyDES components studied in this work are shown in Scheme 1.

**Scheme 1:**
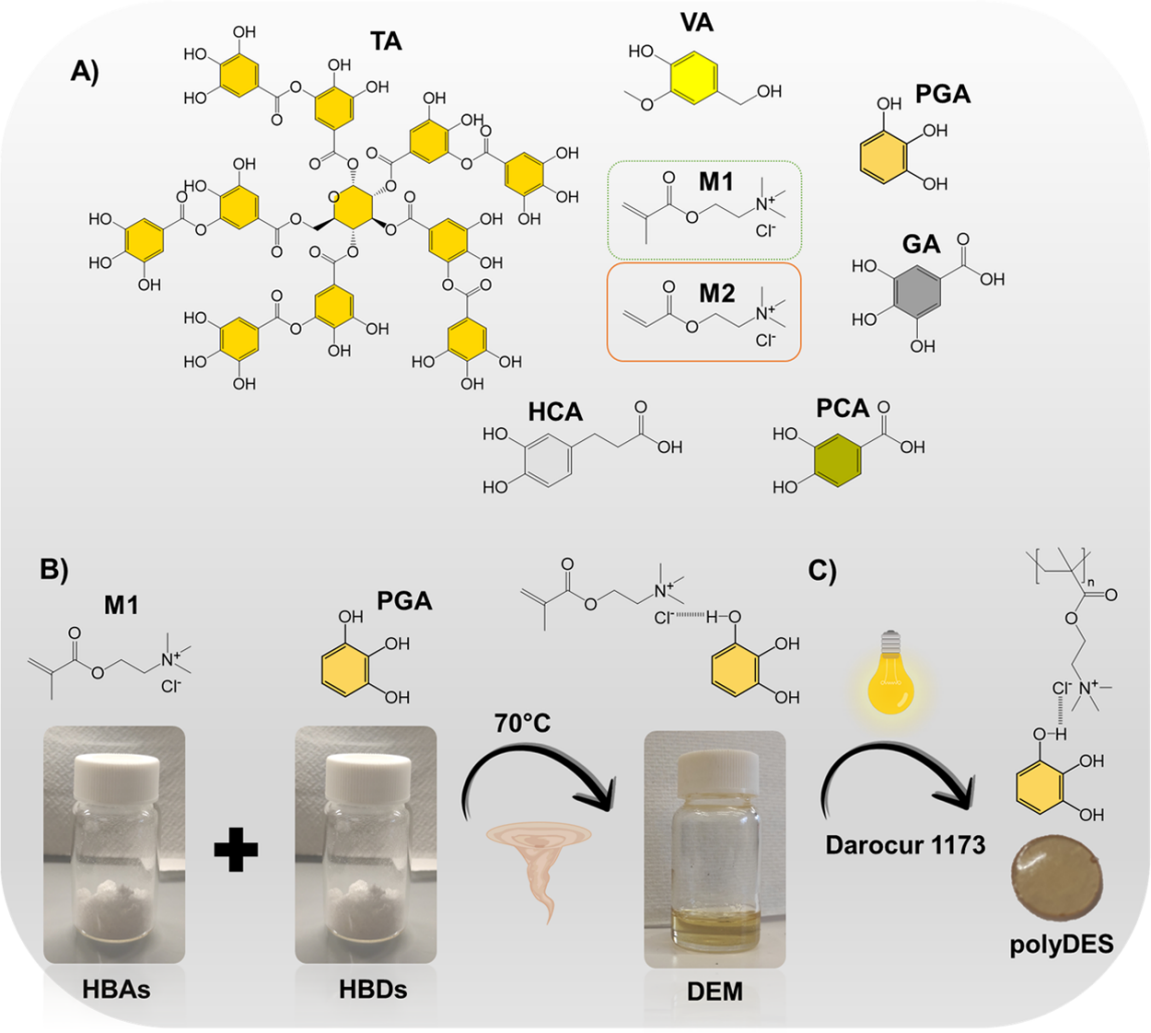
A) Structure of Polyphenols and Quaternary Ammonium Monomers Used for DEM Preparation, B) Schematic Representation of the DEM Preparation Procedure, and C) Photopolymerization Step of DEMs to Produce Phenolic PolyDES

The simple heating/solvent drying method of DES chemistry was used to obtain phenolic DEMs, as shown in Scheme 1B. [2-(Methacryloyloxy) ethyl] trimethylammonium chloride (M1) or [2-(acryloyloxy) ethyl] trimethylammonium chloride solutions (M2) were mixed with various polyphenols at 70 °C under stirring until getting a clear solution. After freeze-drying, the successful formation of the DEMs was proved by differential scanning calorimetry (DSC) and Fourier transform infrared spectroscopy (FTIR) analyses. Table 1 summarizes the different HBD:HBA molar ratios explored to prepare the DEMs and the thermal properties of the obtained mixtures.

**Table 1:** Main Prepared DEMs and Their Thermal Properties

HBD	HBA	HBD:HBA^a^	*T*_g_ (°C)	*T*_d5%_ (°C)	*T*_d__max_ (°C)	Aspect
TA	M1	1:20	–32.2	176.1	255.9	Highly viscous brownish liquid
	M2	1:20	–13.7	152.7	241.7	
GA	M1	1:3^b^	–	–	–	Viscous transparent liquid
	M2	1:3	–20.5	203.6	250.6	
PCA	M1	1:2	–48.8	79.3	262.7	Viscous, yellowish liquid
	M2	1:2	–31.0	193.1	257.1	
HCA	M1	1:2	–40.4	99.2	289.1	Low-viscosity yellowish liquid
	M2	1:2	–30.7	148.0	255.7	
PGA	M1	1:1	–24.5	196.6	272.0	Transparent liquid. Low viscosity
	M2	1:1	–38.1	139.9	303.6	
VA	M1	1:1	–6.7	140.7	263.0	Transparent liquid
	M2	1:1	–16.8	180.3	291.6	

a Note that molar ratios where the mixtures were found to be liquids at room temperature do not certainly correspond to the eutectic compositions.

b The DEM was not stable, showing phase separation after a few days.

The second heating cycle is presented for all DSC experiments. Interestingly, all DEMs showed a glass transition temperature (*T*_g_) instead of a melting point, behaving as low-transition-temperature mixtures (LTTMs) and thereby suggesting the existence of strong interactions between the HBDs and the HBAs. As an illustrative example, DSC curves of PGA-based DEMs are presented in Figure 1A. The DEMs’ *T*_g_ varied depending on the polyphenol structure from −48.8 to −6.7 °C for PCA-M1 (1:2) and VA-M1 (1:1), respectively. In addition, the DEMs’ *T*_g_ could also be modulated by changing the HBD:HBA molar ratio (see Table S1 in the SI). In general, for the same HBD:HBA molar ratio, lower *T*_g_ values were obtained with M1 (methacrylic monomer) compared to M2 (acrylic monomer), except for PGA- and VA-based DEMs. Although further studies are required to understand this behavior deeply, we hypothesized that the polyphenols and the acrylic monomer have a higher affinity, leading to stronger interactions and increasing the glass transition.

**Figure 1:**
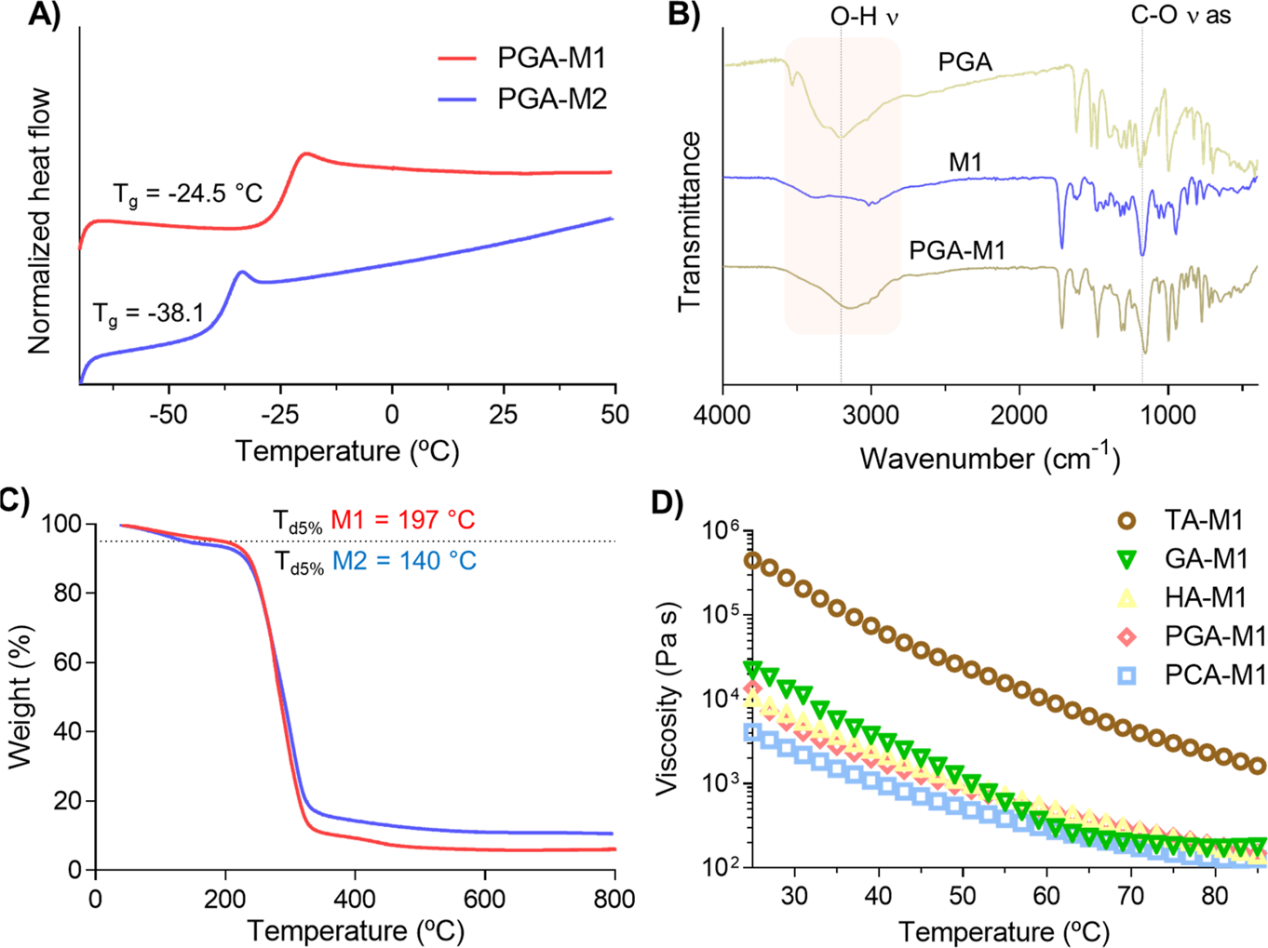
A) DSC curves and *T*_g_ values for PGA-based DEMs (1:1). B) FTIR analysis for PGA, M1, and PGA-M1 DEM. C) Thermal stability of the PGA-based DEMs. D) Evolution of viscosity vs temperature for DEMs based on methacrylic ammonium monomers.

Indeed, FTIR analysis revealed good interaction between the HBA monomers and all the phenolic HBDs evaluated. For instance, spectra of PGA, M1, and the PGA-based DEM are presented in Figure 1B. As observed, the characteristic band of PGA at 3600–2800 cm^–1^ (O–H ν) became broader and shifted from 3205 to 3135 cm^–1^ after DEM formation. Furthermore, the asymmetric C–O ν peak belonging to M1 significantly shifted from 1177 to 1155 cm^–1^ in PGA-M1 DEM. These changes in the characteristic vibrational modes of the pure compounds suggest the constitution of robust hydrogen bonding interactions, probably responsible for suppressing the mixtures’ melting point.

Moreover, the DEMs showed good thermal stability with decomposition temperature values of 5% weight loss (*T*_d5%_) and maximum decomposition temperatures (*T*_dmax_) ranging from 79 to 204 °C and 242–304 °C, respectively (Table 1). The lowest *T*_d5%_ value corresponds to PCA-M1 (1:2), while the highest was obtained for GA-M2 (1:3). It is worth noting that *T*_d5%_ values lower than 100 °C could be associated with water loss due to the highly hygroscopic nature of these eutectic monomers. As a representative case, Figure 1C exhibits the TGA curves obtained for PGA-based samples and corresponding values of *T*_d5%_.

Then we analyzed the temperature effect on the viscosity of the DEMs (Figures 1D and S1). All the mixtures presented similar rheological profiles where the viscosity decreased with the temperature. Curiously, M2-based DEMs presented higher viscosities for all the polyphenols evaluated, probably because stronger HBA–HBD interactions increase the DEMs’ flow resistance. Furthermore, we found that the higher the functional density of the phenolic molecule, the higher the mixture viscosity, probably because the establishment of multiple hydrogen bonding interactions is promoted. As a result, TA-based DEMs showed the highest viscosity.

After obtaining the phenolic DEMs, we proceeded with their photopolymerization to produce a set of functional ionic polyDES (Scheme 1C). Figures 2A and B show the ^1^H NMR spectra of PGA-M1 before and after light irradiation for a few seconds. As can be observed, the vinyl proton signals at 5.6–6.0 ppm in PGA-M1 completely disappear in poly(PGA-M1), revealing complete monomer conversion and excellent efficiency of the photopolymerization process. In addition, a slight shift in the methyl peak of the quaternized nitrogen at around 3.0–3.3 ppm anticipates strong intramolecular forces in the polyDES. FTIR analysis also supported the obtention of the polyDES. As shown in Figure 2C, a band broadening and a hypsochromic shift of the O–H ν (3000 cm^–1^) and C=O ν (1716 cm^–1^) were observed for poly(PGA-M1), probably because of multiple interactions along the polymer backbone. However, the disappearance of the C=C ν band (1615 cm^–1^) after polymerization was unclear, as it could overlap with the ar C–C vibrations of the phenolic molecule (1620 cm^–1^). Furthermore, we observed significant changes in =CH δ ip (1315 cm^–1^), C–O ν (1155 cm^–1^), and =CH δ oop (650 cm^–1^) signals, attributed to the polyDES formation. Regardless of the polyphenol type, the obtained eutectic polymers were water-soluble and slightly hygroscopic (Figure S2).

**Figure 2:**
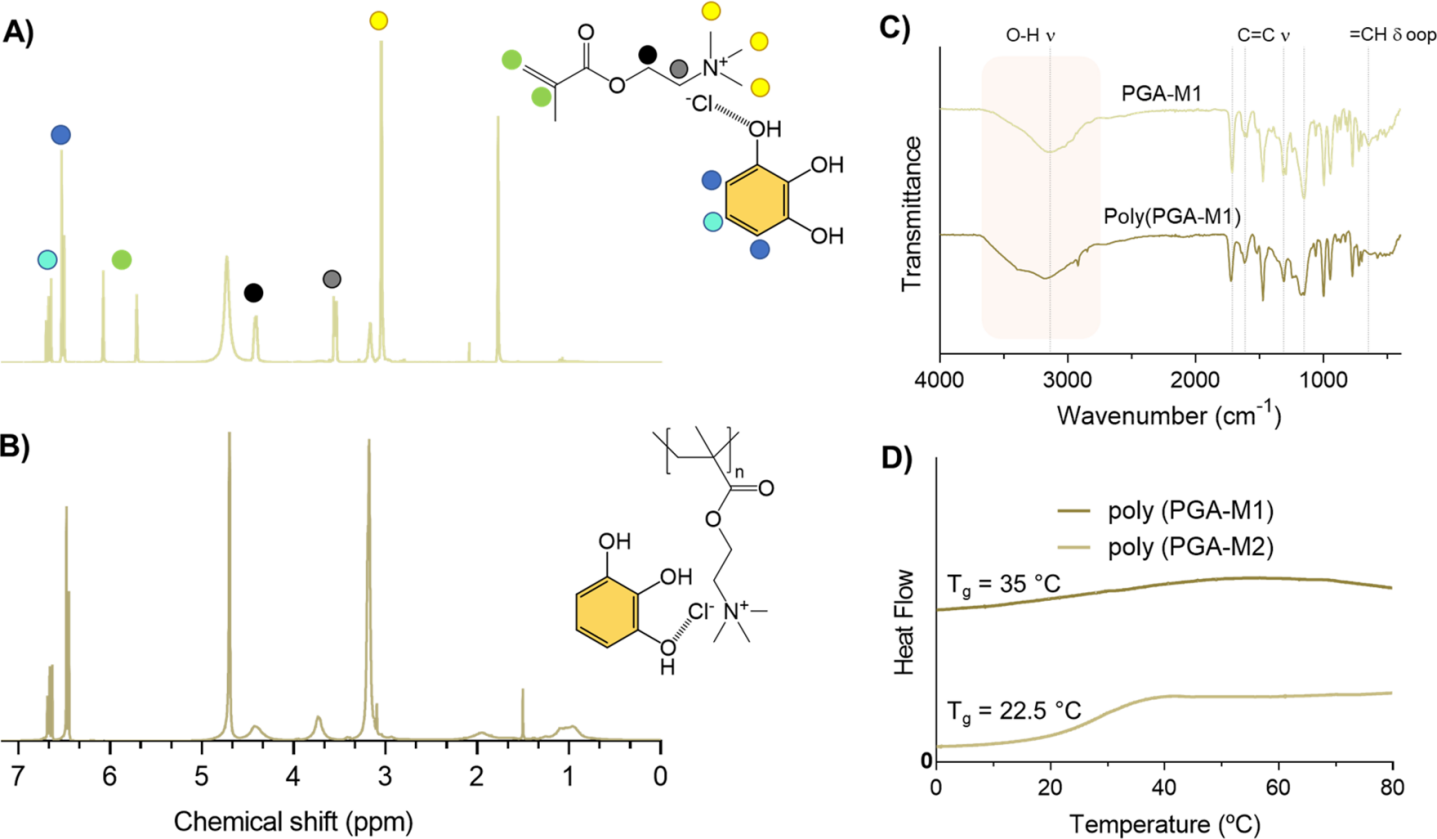
A) ^1^H NMR spectra of PGA-M1 and B) poly(PGA-M1). C) FTIR spectra and D) DSC curves for poly(PGA-M1) and poly(PGA-M2).

We carried out DSC analyses for the phenolic polyDES, finding that the *T*_g_ of these materials seems to be affected by both the type of the HBA (M1 or M2) and the degree of functionality of the polyphenols (Figure S3). For example, TA-based polyDES showed very high *T*_g_ values above 100 °C due to the great number of catechol (5 units) and pyrogallol (5 units) groups in the phenolic molecule, allowing for multiple hydrogen bond interactions. These materials were brittle and nondeformable, whereas other polyDES, such as those from PGA and VA, resembled very viscous gums showing stretchable and adhesive properties. DSC curves of poly(PGA-M1) and poly(PGA-M2) are shown in Figure 2D, evidencing a *T*_g_ of ≈33 and 22 °C, respectively.

Small amplitude oscillatory shear (SAOS) was used to investigate the viscoelastic properties of the novel phenolic polyDES. As expected, amplitude sweeps showed that multifunctional TA led to fragile viscoelastic solids (storage modulus, *G*′ = ≈2 × 10^7^) with an extremely short linear viscoelastic range (<0.1%) (Figure S4A). On the contrary, the rest of the polyDES behaved like viscoelastic liquids with a loss modulus (*G*″) slightly higher than *G*′ in the whole strain range evaluated. For instance, the dynamic moduli vs strain curve for poly(PGA-M1) is displayed in Figure S4B. This behavior is typical of un-cross-linked amorphous polymers (above their *T*_g_) in the transition or terminal zones. Indeed, frequency sweeps for poly(PGA-M1) (Figure 3A) showed a crossover point in the range of 0.1–100 rad/s, where the material behaves like a viscoelastic solid (*G*′ > *G*″) at high frequency. Meanwhile, poly(TA-M1) featured a frequency-independent behavior of *G*′ and *G*″ typical for cross-linked polymers (Figure 3B). Overall, the viscoelastic properties of the polyDES are strongly influenced by the polyphenol type, where small-sized and multifunctional molecules can lead to liquid-like or solid-like materials, respectively.

**Figure 3:**
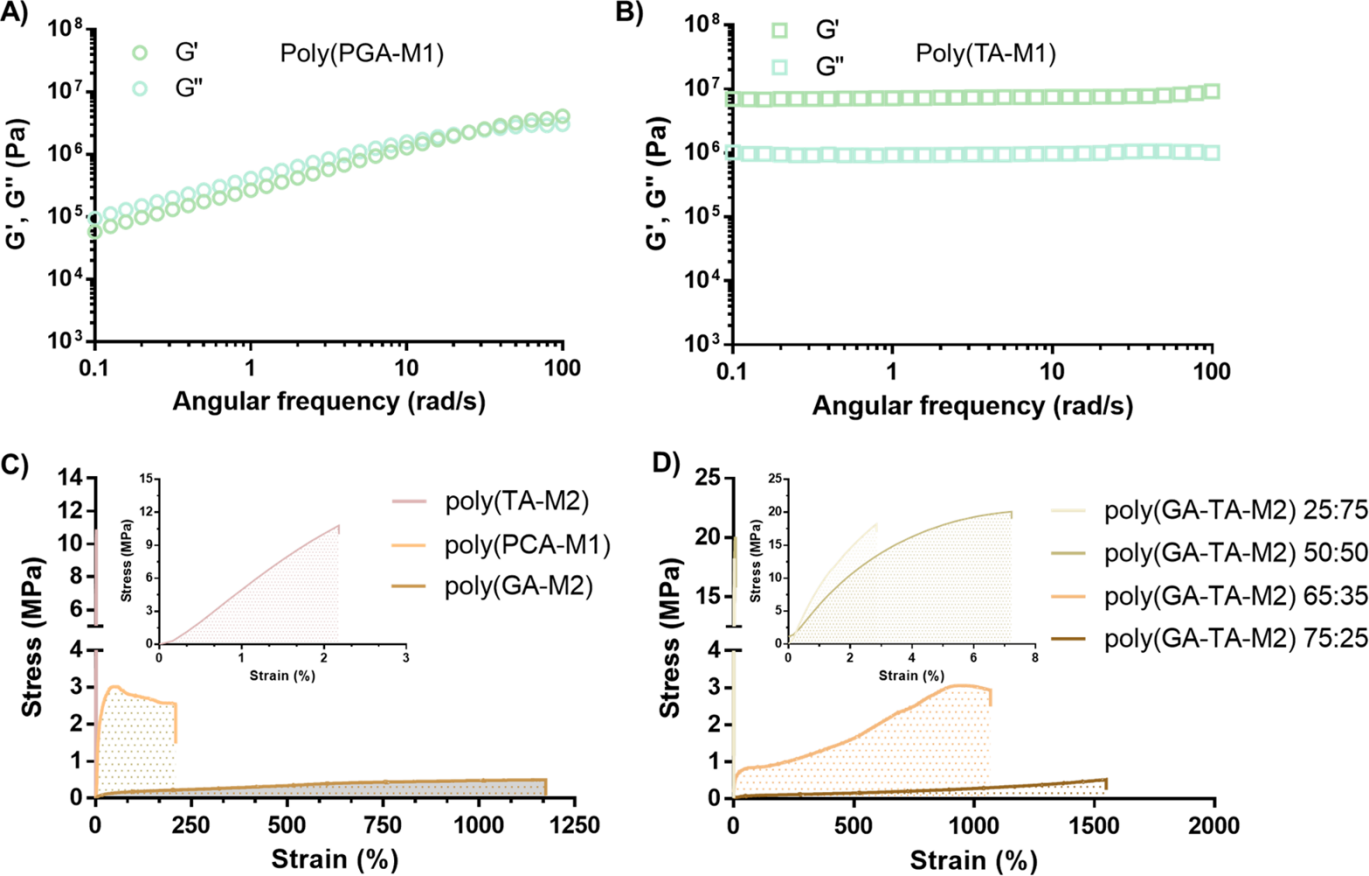
A) Frequency sweep for poly(PGA-M1) and B) poly(TA-M1). C) Strain vs stress curves for TA-, PCA-, and GA-based polyDES. D) Strain vs stress curves for deep eutectic copolymers based on GA and TA.

In this vein, most polyDES were too soft after polymerizing, and only four formulations afforded self-standing materials: poly(TA-M1), poly(TA-M2), poly(PCA-M1), and poly(GA-M2). Therefore, we further studied their mechanical properties by a tensile test, and the results are presented in Figure 3C. It is worth mentioning that poly(TA-M1) was too brittle to be tested and was excluded from this study. Similarly, due to the dense distribution of hydrogen bonding in the TA, poly(TA-M2) can only withstand small deformations of ≈2% with tensile strength as high as 11 MPa (inset Figure 3C). On the other hand, poly(PCA-M1) and poly(GA-M2) were flexible materials showing elongation at break values of 210 and 1175% and strengths of 3 and 0.5 MPa, respectively. Evidently, the molecular structure of the polyphenol can regulate the hydrogen bonding density in the polyDES, which in turn dominates the mechanical performance of these materials. Indeed, when analyzing Young’s modulus of the materials, it decayed from 525 to 0.17 MPa for poly(TA-M2) and poly(GA-M2).

Therefore, we wonder if the stiffness and stretchability of these ionic polyDES could be modulated by producing copolymers from the brittle TA and soft GA-based formulations, which were utterly miscible. As displayed in the inset of Figure 3D, mixtures of GA:TA = 25:75 and 50:50 wt % ratio only slightly improved the extensibility of the materials compared to poly(TA-M2). However, when increasing the content of the soft DEM up to 65:35 and 75:25, a radical increase in the stretchability and toughness of these polyDES was reached. For instance, poly(GA-TA-M2) 65:35 resulted in an ultratough elastomer (20 MJ/m^3^) with a maximum elongation of 1070% and a strength of 3 MPa. Further characterization of this outperforming copolymer, including ^1^H NMR, FTIR, and DSC analysis, is presented in the SI (Figure S5).

Catechol and pyrogallol groups in the phenolic HBD are well-known chemical motifs responsible for the extraordinary underwater adhesion of several marine organisms like mussels and ascidians.^26,27^ Therefore, HCA- and PGA-based polyDES showing viscoelastic liquid behavior are excellent candidates to be explored as adhesives. We found that methacrylate-based formulations of these polyphenols present poor adhesive properties, as shown in Figures S6A,B. On the other hand, the acrylate-based HCA and PGA polyDES resulted in extremely sticky materials with adhesive stresses around 1.25 MPa (Figures 4A,B). Indeed, the excellent adhesive properties of poly(PGA-M2) allowed holding a water-filled flask and a vial joined while lifting the pieces of around 0.5 and even up to 1.6 kg (Figures 4C and S6C).

**Figure 4:**
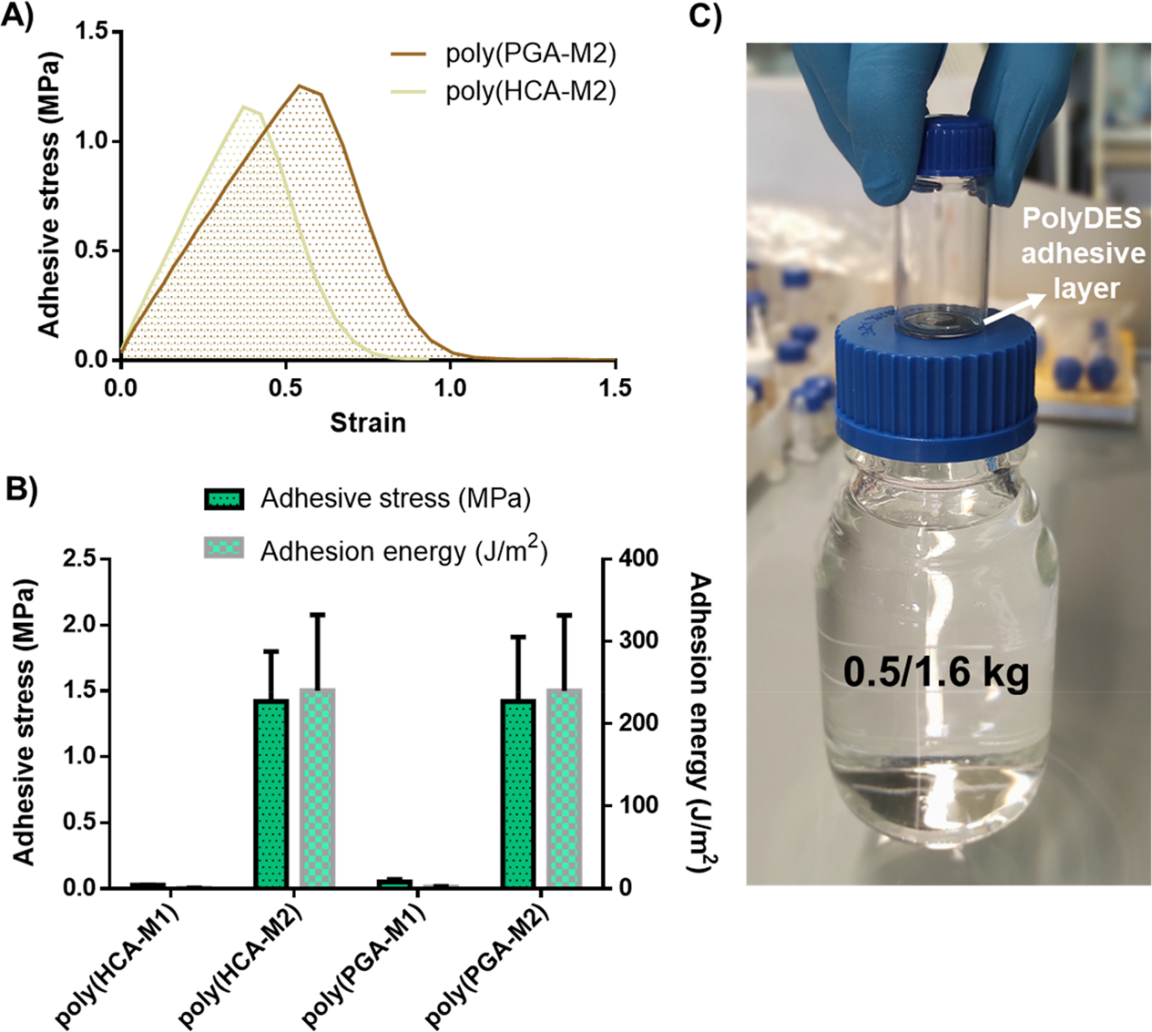
A) Adhesive stress vs strain curves for poly(PGA-M2) and poly(HCA-M2). B) Adhesive stress vs adhesive energy for both adhesive polyDES. C) Photo of a water-filled flask and a vial joined by a poly(PGA-M2) adhesive layer while lifting the piece of around 0.5 kg.

Since poly(TA-M2) and poly(PGA-M2) exhibited well-distinctive stiff and soft features, we investigated the molecular weight (*M*_w_) of these polyDES as it could affect their behavior beyond the polyphenol structure. Both polyDES showed broad molecular weight distributions with *M*_n_ and polydispersity values of 9.9 kDa and 5.3 for poly(TA-M2) and 66.1 kDa and 3.2 for poly(PGA-M2) (Figure S7).

Besides their adhesive features, catechol and pyrogallol groups stand out for their metal ion coordination ability, and for this reason, natural polyphenols have drawn increasing interest as promising platforms for water remediation devices.^28−30^ Thus, as a proof of concept, we choose the multifunctional TA-based methacrylic polyDES to evaluate its ion complexation capacity, employing iron (III) as a metal model. In addition, since phenolic polyDES are highlighted by a fast and complete monomer conversion, they are particularly attractive for digital light processing 3D printing, where different objects, such as a flower, could be easily manufactured (Figure 5A). Hence, we patterned poly(TA-M1) into a honeycomb-like scaffold with 1 mm pores (Figure 5B), aiming to increase the metal absorption after immersing this material in an iron chloride solution of TRIS buffer (pH 8). After the immersing period, the scaffold became utterly dark (Figure 5C), suggesting the formation of a tris complex (inset Figure 5D) between the iron and the polyphenol.^31,32^ Indeed, UV analysis revealed that the characteristic peak of TA at 275 nm (Figure S8) shifted to 535 nm because of the iron coordination.

**Figure 5:**
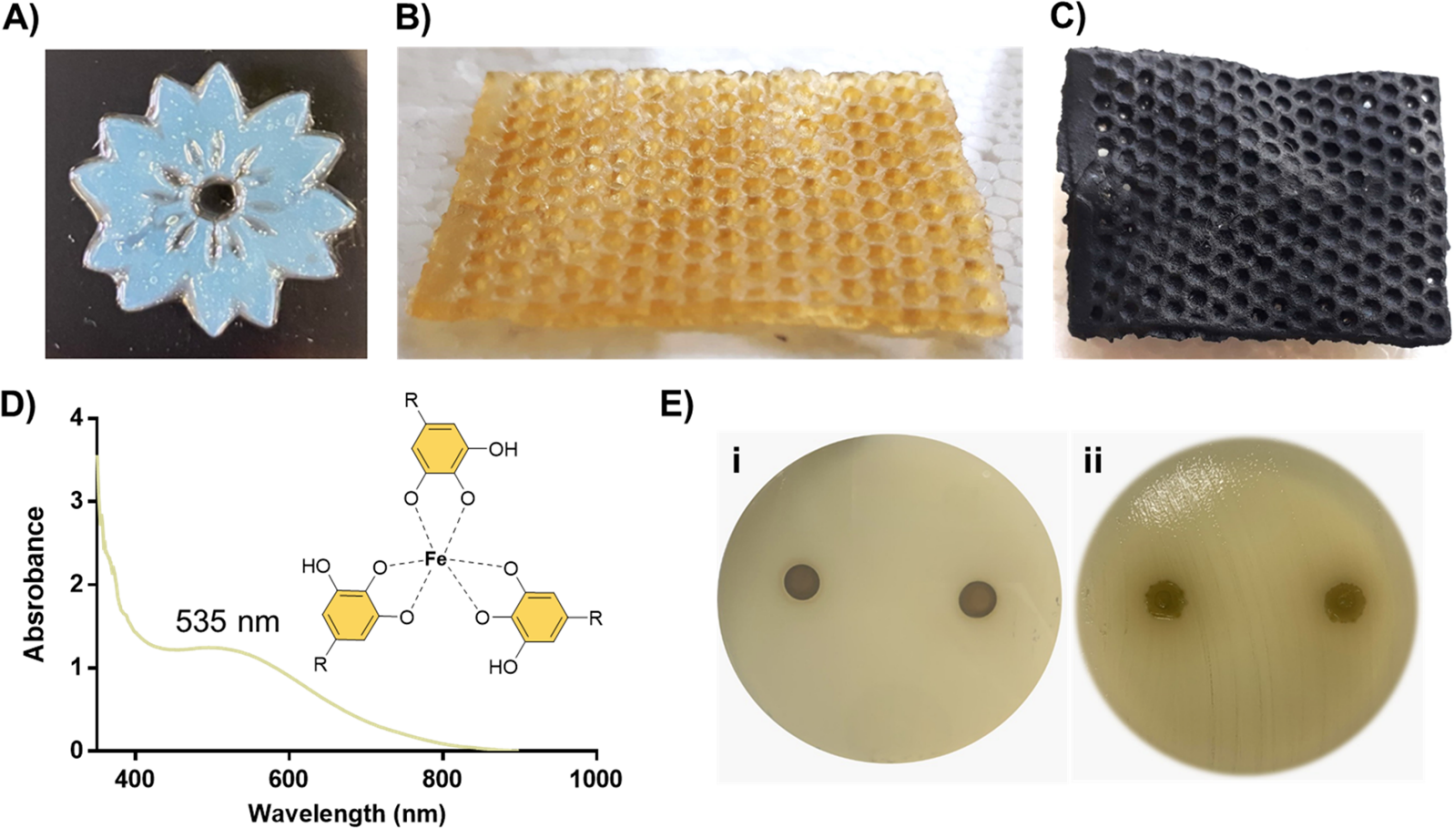
A) Photo of a flower manufactured by digital light processing 3D printing from GA-M2 ink. **B**) Photo of poly(TA-M1) 3D-patterned into a honeycomb-like scaffold with 1 mm pores. C) Photo of poly(TA-M1) scaffold after iron coordination. D) UV–vis spectrum of the TA-Fe(III) complex released from poly(TA-M1) after immersion in 0.02 M FeCl_3_ TRIS buffer solution (pH 8). E) Antibacterial activity of poly(TA-M1) against *Escherichia coli* after 18 h of incubation. (i): Plate with the discs. (ii): Plate without the discs.

Finally, these innovative polyDES could benefit from the polyphenols’ therapeutic properties, turning them into attractive materials for biomedical applications.^33,34^ As an example, we explored the antibacterial activity of poly(TA-M1) against *Escherichia coli* by an agar diffusion test. As shown in Figure 5E, no inhibition zone was observed after 18 h of incubation (left image (i)); however, after disc removal, no bacteria growth was evidenced in the area where the materials were deposited (right image (ii)). Since TA is a well-known antimicrobial agent,^35^ these results suggest that their diffusion from the polyDES is quite limited due to strong intermolecular interactions, restricting its antibacterial activity to the contact zone.

This letter identified an innovative family of phenolic deep eutectic monomers for designing ionic polymers with multiple functionalities. Natural phenolic compounds bearing catechol or pyrogallol groups showed an excellent affinity with quaternary ammonium monomers, giving liquid eutectic mixtures with complete suppression of their melting points. The resulting (meth)acrylic deep eutectic monomers could be easily photopolymerized. The properties of the obtained ionic polyDES could be tuned, by selecting suitable phenolic molecules, from ultratough materials with stretchabilities over 1000% and strengths around 3 MPa, to superadhesive viscoelastic liquids. Catechol and pyrogallol motifs endow these polyDES with various functional properties like extraordinary adhesion, metal complexation ability, antibacterial activity, and potential redox capacity. All in all, this study represents the first example where polyphenol chemistry is brought to the innovative deep eutectic monomers realm, allowing expanding its functionality and range of applications.

## Supporting Information Available

Materials and methods; *T*_g_ of DEMs prepared with different HBD:HBA molar ratios; viscosity vs temperature for phenolic DEMs based on M1 and M2 monomers; water uptake of the polyDES; *T*_g_ for the obtained polyDES; amplitude sweeps for poly(TA-M1) and poly(PGA-M1) polyDES; ^1^H NMR, FTIR, and DSC analyses for poly(GA-TA-M2) 65:35; adhesive stress vs strain curves for poly(PGA-M1) and poly(HCA-M1); molecular weight distribution of poly(TA-M2) and poly(PGA-M2); and UV spectrum of TA in water (PDF)
