# Supplementary material for: Multifunctional Ionic Polymers from Deep Eutectic Monomers Based on Polyphenols
Source: ACS Macro Lett. 2023 Jan 12;12(2):125–32. doi: 10.1021/acsmacrolett.2c00657 (PMC9948532; doi:10.1021/acsmacrolett.2c00657)
Supplement: Supplementary file 1 — mz2c00657_si_001.pdf [file mz2c00657_si_001.pdf]

Supporting Information

Multifunctional ionic polymers from deep eutectic monomers based on polyphenols

Jon López de Lacalle,[1] Antonela Gallastegui,[1] Jorge L. Olmedo-Martínez,[1] Melissa Moya,[2,3] Naroa Lopez-Larrea,[1] Matías L. Picchio,[1,4*] David Mecerreyes[1,5*]

[1] POLYMAT and Department of Polymers and Advanced Materials: Physics, Chemistry and Technology, Faculty of Chemistry, University of the Basque Country UPV/EHU, Paseo Manuel de Lardizábal, 3, 20018 Donostia-San Sebastián, Spain.

[2] Laboratorio de Investigación, Universidad de Ciencias Médicas, San José, Costa Rica.

[3] Facultad de Microbiología, Universidad de Ciencias Médicas, San José, Costa Rica.

[4] Instituto de Desarrollo Tecnológico para la Industria Química (INTEC), CONICET, Güemes 3450, Santa Fe 3000, Argentina.

[5] IKERBASQUE, Basque Foundation for Science, Plaza Euskadi 5, 48009, Bilbao, Spain.

Corresponding authors:

Matías L. Picchio (E-mail: mlpicchio@santafe-conicet.gov.ar)

David Mecerreyes (E-mail: david.mecerreyes@ehu.es)

Experimental section

Materials

[2-(Methacryloyloxy) ethyl] trimethylammonium chloride solution (M1, 75 wt.% in water), [2-(acryloyloxy) ethyl] trimethylammonium chloride solution (M2, 80 wt.% in water), 2-hydroxy-2-methylpropiophenone (Darocur 1173, 97%), poly(ethylene glycol) diacrylate (PEGDA Mw= 550,), tannic acid (TA, ACS reagent), gallic acid (GA, 97,5-102,5% titration), 2,4-dihydroxyhydrocinnamic acid (HCA, 98%), pyrogallol (PGA, >98%), 4-hydroxy-3-methoxybenzyl alcohol (VA, 98%), copper(II) chloride (97%), cobalt(II) chloride (97%), and iron(III) chloride (97%) were purchased from Sigma-Aldrich. 3,4-dihydroxybenzoic acid (PCA, 97%) was supplied by Alfa Aesar. All chemicals were used without further purification.

Preparation of deep eutectic monomers (DEMs)

DEMs were prepared by mixing the ammonium salt monomer dissolved in water (75-80 wt.%) with the different polyphenols at defined molar ratios under stirring at 70°C. After a homogeneous liquid was obtained, the mixture was freeze-dried for two days for water elimination. Different HBD: HBA molar ratios were evaluated, and only those mixtures that remained in a liquid state at room temperature after freeze-drying are reported.

Synthesis of polymeric deep eutectic solvents (PolyDES)

PolyDES were obtained by photopolymerizing DEMs mixture using Darocur 1175 (5 wt%) as the photoinitiator. DEMs were poured into silicone molds and UV-irradiated three times in a Light Curing Systems UVC-5 (Dymax) with a lamp intensity of 400 mW·cm^{-2}. After a solid material was obtained, the sample was peeled off and stored at room temperature for posterior characterization.

Characterizations

Thermal analysis

The thermal properties of the DEMs and polyDES were analyzed by thermogravimetric analysis (TGA) and differential scanning calorimetry (DSC). TGA analysis was carried out on a PerkinElmer Thermogravimetric Analyzer TGA 8000. Samples of about 15 mg were heated from 30 to 800 °C at a heating rate of 10 °C·min^{-1} and under a nitrogen atmosphere. The temperature at the maximal decomposition rate (T_{max}) was determined at the main peak of the derivative weight loss curve.

The DSC experiments were performed on a PerkinElmer 8000 DSC equipped with an Intracooler II. Samples of roughly 3 mg were crimped in non-recyclable aluminum hermetic pans and analyzed under a nitrogen atmosphere by heating and cooling cycles at 20 °C·min^{-1}. First, the samples were heated from 25 to 150 °C and kept isothermally for 3 min to erase the thermal history. Subsequently, the samples were cooled to –70 °C and kept isothermally for 3 min. Second-run heating cycles were conducted and further used to investigate the phase transition behavior of all samples.

FITR spectroscopy

Fourier transform infrared (FTIR) spectra were recorded on a Bruker Alpha II spectrophotometer employing a Platinum ATR module with a diamond window.

Hygroscopicity

The humidity uptake of the polyDES was evaluated by following their weight change over time when exposed to the open air at room temperature for 7 days. Results are expressed as weight gain in percentage.

NMR spectroscopy

^{1}H nuclear magnetic resonance (NMR) spectra were recorded in a Bruker Avance DPX 300 at 300.16 MHz, using deuterated dimethylsulfoxide (DMSO-d6) as solvent at room temperature.

Gel permeation chromatography (GPC)

GPC was employed to determine the molar mass of the polyDES using a Waters device with three Ultrahydrogel columns (2000, 200, 120 Å). The mobile phase was 0.1 M NaCl/ 0.1% trifluoroacetic acid, the flow rate 0.6 $mL \cdot min^{-1}$, and the sample concentration 2 $mg \cdot mL^{-1}$. The molar masses were referred to poly(ethylene glycol) standards (1470-1039000 Da).

Rheological measurements

All the experiments were performed in a stress-controlled Anton Paar Physica MCR101 rheometer. Temperature-dependent viscosities were measured from 25 to 90°C at a fixed shear rate of 50 s^{-1} using a 25 mm cone-plate geometry. Amplitude sweeps were carried out from 0.1 to 100% strain at a constant frequency of 1 Hz at 25 ºC. Frequency sweeps were performed from 0.1 to 100 rad $\cdot s^{-1}$ at 1% strain at 25°C. Temperature sweep experiments were carried out from 0 to 80°C at 4°C$\cdot min^{-1}$ and a fixed frequency of 1 Hz and 0.1% strain (in the linear range of viscoelasticity of the materials). An 8 mm parallel-plate geometry was used for amplitude, frequency, and temperature experiments.

Tensile test

For the tensile tests, gel specimens with bone shapes of 25 mm in length and cross-section 3.5 mm × 1 mm were prepared. Tests were carried out using a TA HD plus Texture Analyzer equipment (Stable Micro Systems) at 23 °C, 50%

relative humidity, and an elongation rate of 25 mm·min^{-1}. At least five experiments of each sample were carried out.

Probe-tack test

Adhesion measurements were performed using a TA HD plus Texture Analyzer equipment (Stable Micro Systems). Samples were prepared on glass support by applying an adhesive layer of about 250 μm. In the probe-tack tests, a circular Delrin® probe (10 mm Ø) comes into contact with the sample at a given velocity of 1 mm·s^{-1}. A 500 g compressive force is applied for 1 s, and the probe is removed from the film at a controlled velocity. The debonding force and displacement were recorded and then converted to nominal stress and strain by normalizing the force by the probe area and the displacement by the initial thickness of the gel, respectively.

The material stickiness was defined as the maximum stress, while the tack adhesion energy (W_{adh}) was calculated from the area under the stress-strain curve and the sample thickness.

Digital Light 3D-printing

The photopolymerizable DEMs inks were placed into the vat of the 3D printer (Phrozen Sonic Mini) and exposed to UV light (wavelength: 405 nm, power: 2 mW·cm^{-2}). Printing patterns were first designed with Autodesk Inventor 2021 software and then printed with 50 seconds irradiation time, 0.2 mm layers height, 7 seconds off delay, and 50 mm·min^{-1} lifting speed.

Metal Adsorption

3D-patterned poly(TA-M1) meshes were immersed in 10 mL of the 0.02 M $FeCl_3$ solution with TRIS buffer at pH 8 for 24 h. After sample removal, the remaining

solution was analyzed on a Shimadzu UV-2550 spectrophotometer to determine the metal-polymer complexation.

Antibacterial activity

The antibacterial activity of the poly(TA-M1) was evaluated by the agar diffusion test on 6 mm discs. Firstly Mueller-Hinton agar plates were prepared, and a bacterial suspension of *Escherichia coli* was prepared using a 0.5 Mc Farland standard in sterile saline. Then it was inoculated in the plates using a sterile swab by streaking it three times over the entire agar surface.

The discs previously sterilized with UV light were placed over the agar surface with the bacteria and incubated for 18 h at 35°C ± 2°C. After incubation, the results of antibacterial activity were analyzed.

Table S1. T_g of DEMs prepared with different HBD: HBA molar ratios.

HBD	HBA	HBD: HBA	T_g (°C)	Aspect
HCA	M1	2: 1	-7.5	Low-viscosity yellowish liquid
		1: 1	-19.0	
	M2	2: 1	-12.1	
		1: 1	-13.6	
PGA	M1	2: 1	-12.4	Transparent liquid. Low viscosity
		1: 2	0.7	
	M2	2: 1	-13.8	
		1: 2	-22.6	

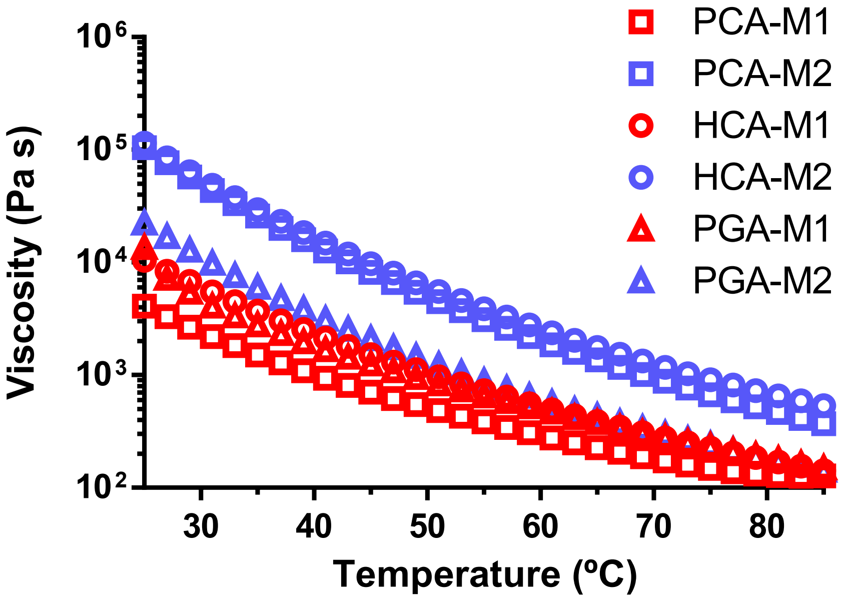

Figure S1. Viscosity variation with temperature for phenolic DEMs based on M1 and M2 monomers.

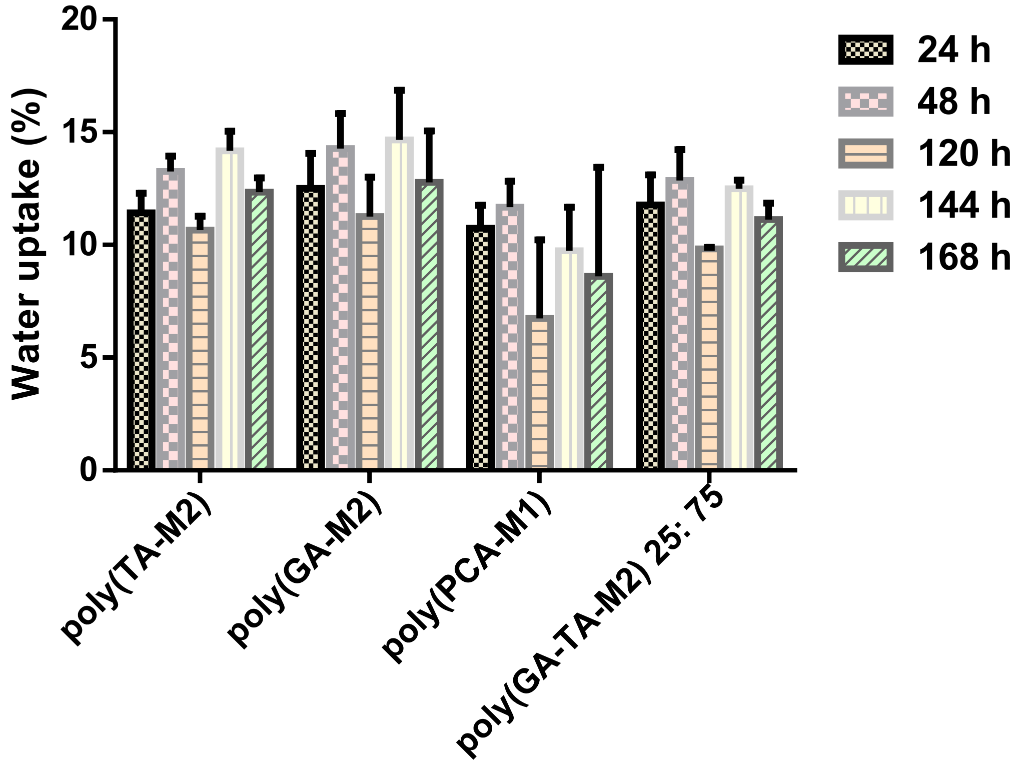

Figure S2. Water uptake of the polyDES when exposed to the open air at room temperature for 168 h.

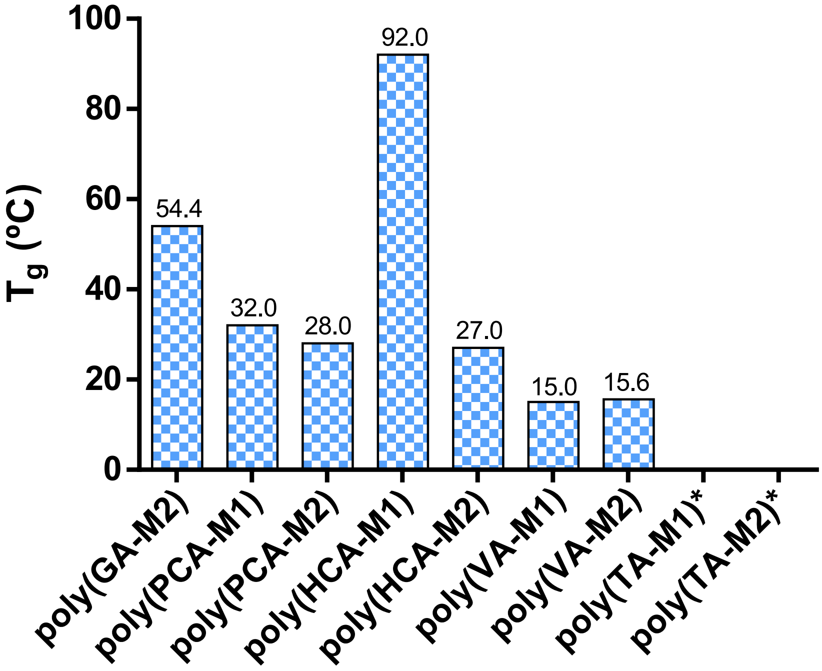

Figure S3. T_g for the obtained polyDES. *A T_g was not detected for TA-based polyDES in the temperature range of -70 –150 °C.

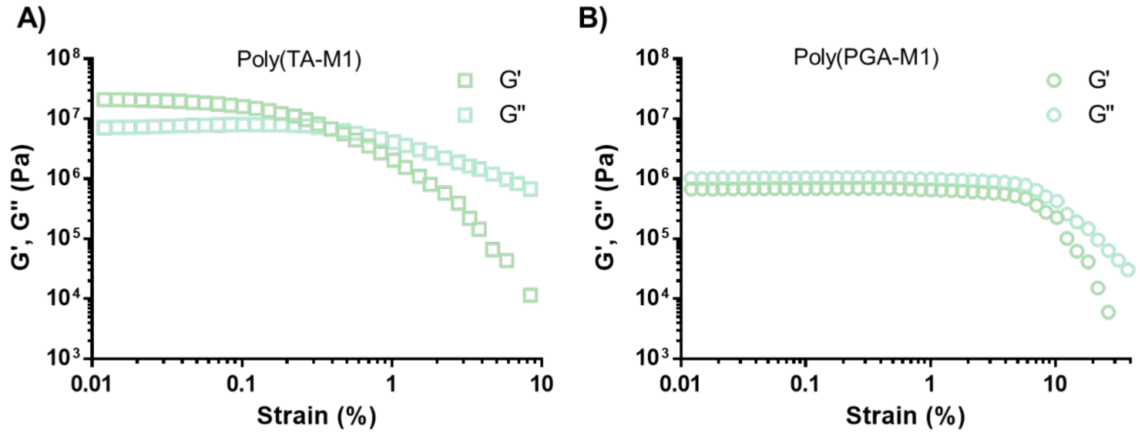

Figure S4. Amplitude sweeps for A) poly(TA-M1) and B) poly(PGA-M1) polyDES.

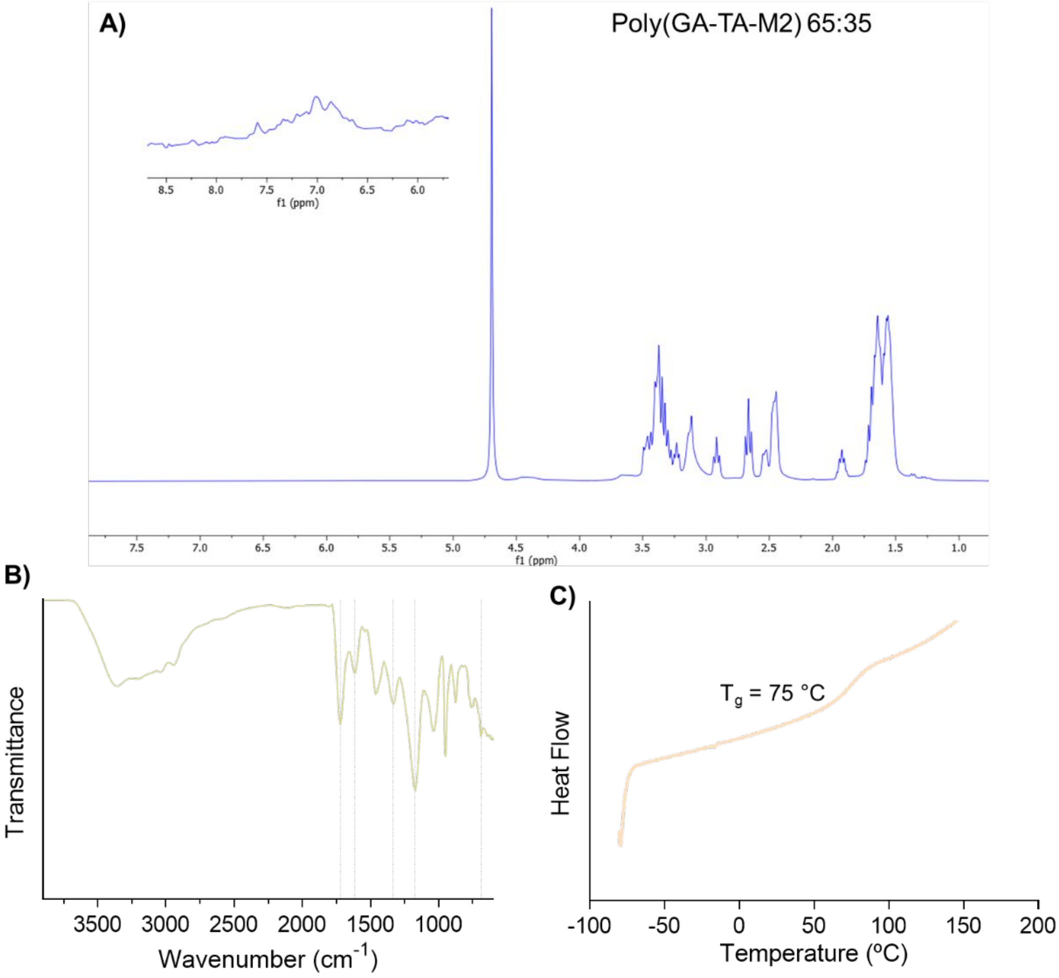

Figure S5. A) ^{1}H NMR, B) FTIR, and C) DSC analyses for poly(GA-TA-M2) 65: 35.

^{1}H NMR spectrum of Figure S4A demonstrated a full monomer conversion in the synthesis of the GA-TA-M2 copolymer as vinyl proton signals at 5.5-7.0 ppm were not present. Furthermore, the characteristic vibrational modes of the polymer and phenolic molecules were evidenced by FTIR at 1721 (C=O ν), 1615 (ar C-C vibrations), 1335 (O-H δ ip), 1175 (C-O ν), and 688 cm^{-1} (O-H δ ip) (Figure S4B). The T_g of the copolymer was around 75°C as determined by DSC analysis (Figure S4C).

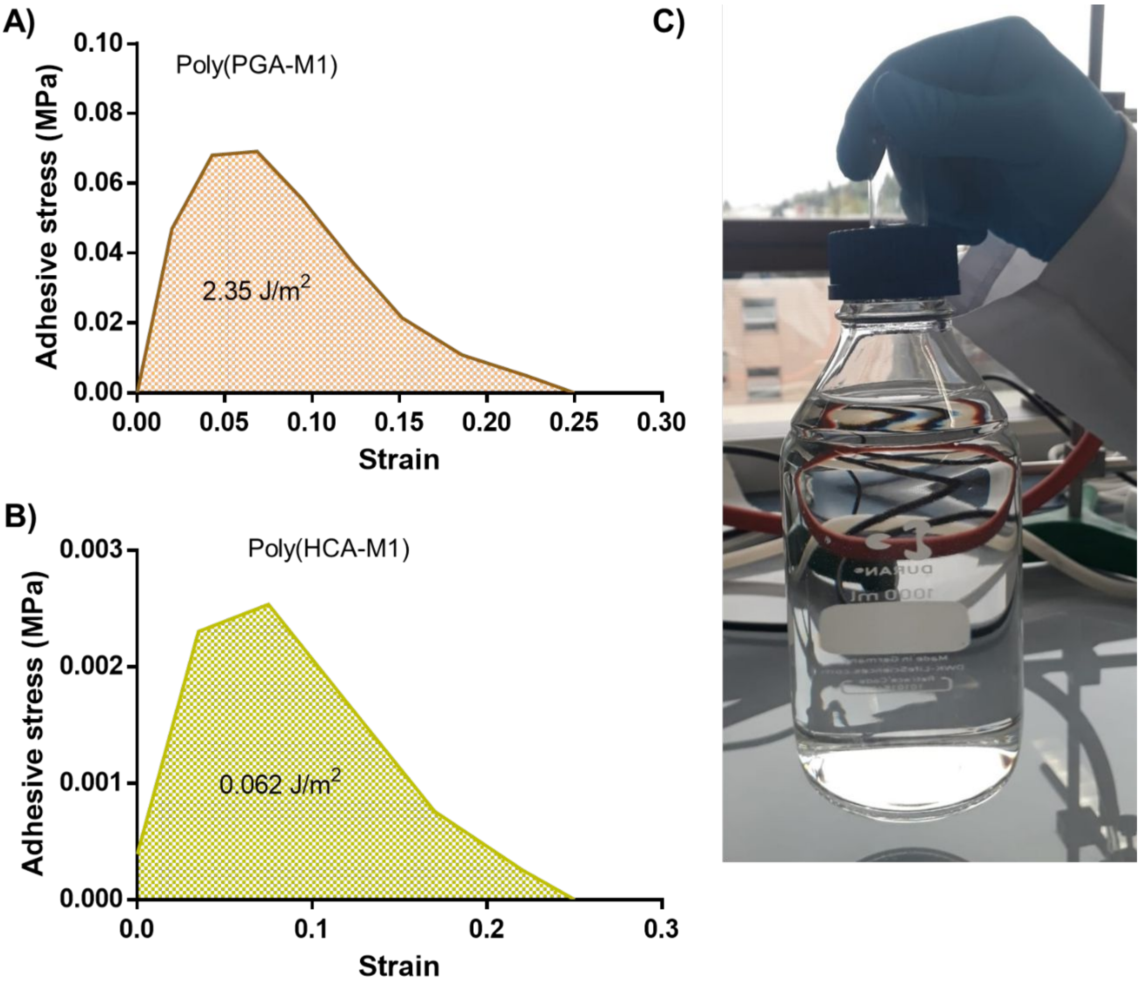

Figure S6. Adhesive stress vs. strain curves for A) poly(PGA-M1) and B) poly(HCA-M1). C) Photo of a water-filled flask and a vial joined by a poly(PGA-M2) adhesive layer while lifting the piece of around 1.6 kg

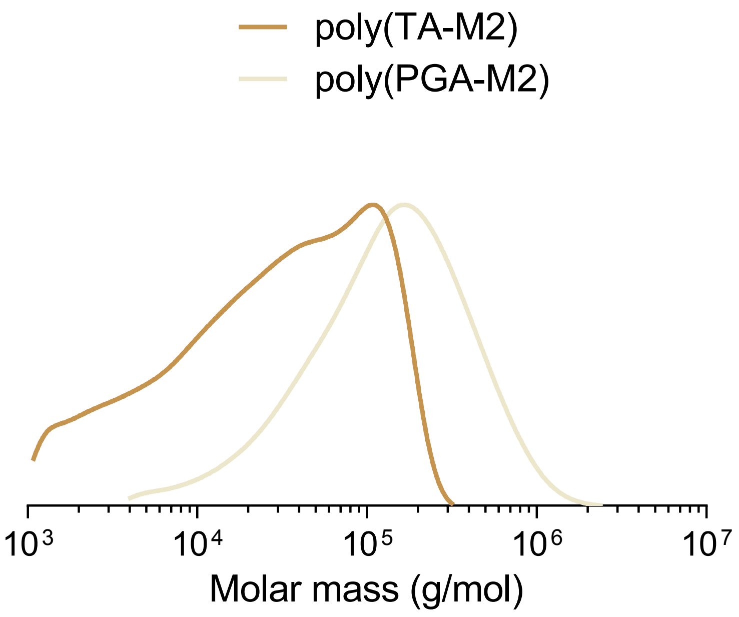

Figure S7. Molecular weight distribution of poly(TA-M2) and poly(PGA-M2) determined by GPC analysis.

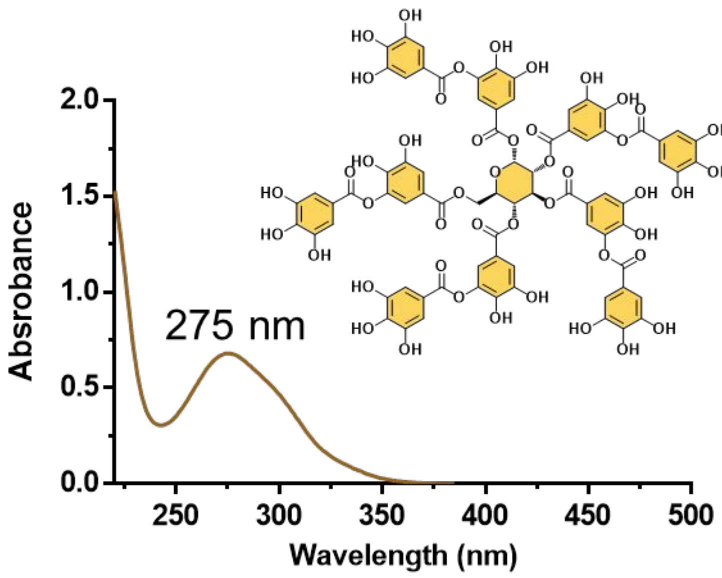

Figure S8. UV spectrum of TA in water.
